# Genetic Modification of *Bergera koenigii* for Expression of the Bacterial Pesticidal Protein Cry1Ba1

**DOI:** 10.3389/fpls.2022.899624

**Published:** 2022-05-24

**Authors:** Seyed Ali Ravanfar, Diann S. Achor, Nabil Killiny, Turksen Shilts, Yuting Chen, Choaa El-Mohtar, Lukasz L. Stelinski, Bryony C. Bonning, Vladimir Orbović﻿﻿﻿

**Affiliations:** ^1^Citrus Research and Education Center, University of Florida, IFAS, Lake Alfred, FL, United States; ^2^Department of Entomology, Iowa State University, Ames, IA, United States; ^3^Entomology and Nematology Department, University of Florida, Gainesville, FL, United States

**Keywords:** *Bergera koenigii*, *Murraya koenigii*, genetic transformation, pesticidal protein, Cry1Ba1, Asian citrus psyllid, Bt toxin

## Abstract

The curry leaf tree, *Bergera koenigii*, is highly attractive to the Asian citrus psyllid, *Diaphorina citri*, which vectors the bacterial causative agent of citrus greening or huanglongbing disease. This disease has decimated citrus production in Florida and in other citrus-producing countries. As *D. citri* exhibits high affinity for feeding on young leaves of *B. koenigii*, transgenic *B. koenigii* expressing bacteria-derived pesticidal proteins such as Cry1Ba1 have potential for *D. citri* management when planted in or adjacent to citrus groves. Importantly, the plant pathogenic bacterium that causes citrus greening does not replicate in *B. koenigii*. Transgenic plants of *B. koenigii* were produced by insertion of the gene encoding the active core of the pesticidal protein Cry1Ba1 derived from *Bacillus thuringiensis*. The transformation success rate was low relative to that of other citrus, at 0.89%. T-DNA integration into the genome and *cry1ba1* transcription in transgenic plants were confirmed. Transgenic plants expressing Cry1Ba1 differed from wild-type plants, differed in photosynthesis parameters and hormone levels in some instances, and a marked delay in wilting of detached leaves. The gut epithelium of *D. citri* fed on transgenic plants was severely damaged, consistent with Cry1Ba1-mediated pore formation, confirming expression of the pesticidal protein by transgenic *B. koenigii*. These results demonstrate that transgenic *B. koenigii* expressing bacteria-derived pesticidal proteins can be produced for potential use as trap plants for suppression of *D. citri* populations toward protection of citrus groves from citrus greening.

## Introduction

Citrus greening or huanglongbing (HLB) has reduced Florida citrus production by >75% since 2003 and has severely impacted other citrus growing regions around the world (Gottwald, 2010; Li et al., 2021). The fruit yield from infected trees is dramatically decreased and infected trees ultimately die. All commercially important cultivars of citrus are susceptible to the “*Candidatus* Liberibacter asiaticus” (CLas) bacterium that causes this disease (Folimonova et al., 2009). As the Asian citrus psyllid (ACP; *Diaphorina citri*) is the presumed vector of CLas (Bove, 2006), citrus growers in Florida relied on repeated applications of chemical insecticides for control of ACP in an attempt to counter disease transmission (Bove, 2006; Tiwari et al., 2012). This practice rapidly selected for insecticide resistance to some of these insecticides in Florida ACP populations (Tiwari et al., 2011). The management of citrus greening requires an integrated and multi-pronged approach targeting both CLas and ACP, and alternative approaches are urgently required to mitigate the impacts of this disease on the citrus industry.

The Indian curry leaf plant, *Bergera koenigii,* is a member of the same plant family as *Citrus* (Rutaceae) but is cultivated for curry leaf rather than fruit production. *B. koenigii* leaves are dried, ground, and used for the production of curry spice. Two important observations were made relating to the curry plant: First, ACP preferred *B. koenigii* over citrus plants in olfactory tests (Beloti et al., 2017), and second, although *B. koenigii* is attractive to ACP, it is not a host for CLas (Beloti et al., 2018). The ability of *B. koenigii* to attract ACP without serving as a reservoir for CLas makes this plant an excellent candidate for use as a trap plant when combined with an insecticidal protein that is toxic to ACP.

Transgenic plant expression of pesticidal proteins derived from *Bacillus thuringiensis* (Bt) and other bacteria (Crickmore et al., 2020) has been successfully used for more than two decades for insect pest management in cotton, corn, rice, and other crops (Shelton et al., 2002; Calles-Torrez et al., 2017; Li et al., 2017; Fleming et al., 2018). While these proteins typically have reduced efficacy against phloem-feeding insects such as ACP without appropriate optimization (Chougule and Bonning, 2012; Chougule et al., 2013), two pesticidal proteins active against ACP have been reported (Fernandez-Luna et al., 2019), with contradicting reports about a third (Fernandez-Luna et al., 2019; Dorta et al., 2020). For the purpose of suppression of ACP populations in citrus groves, a Bt-derived pesticidal protein could be delivered to ACP *via* an alternative ACP host such as Indian curry leaf plant. When planted around or in citrus groves, transgenic *B. koenigii* plants should attract ACP away from citrus trees and deliver the Bt-derived pesticidal protein as the insect feeds. Use of transgenic *B. koenigii* expressing such proteins in citrus groves should suppress ACP populations, lower the cost of management of citrus groves, and benefit the environment through decreased frequency of insecticide application.

The genetic transformation of *B. koenigii* has not previously been reported. We sought to address whether genetic transformation of *B. koenigii* for expression of an ACP-active, bacterial pesticidal protein such as Cry1Ba1 could be achieved. We employed *Agrobacterium*-mediated transformation for integration of the *cry1ba1* gene into the *B. koenigii* genome. Integration of T-DNA, along with transcription and translation of *cry1ba1,* was confirmed. Following observation of a delayed wilting phenotype, changes in photosynthetic parameters and phytohormone levels relative to non-transformed, wild-type plants were also examined.

## Materials and Methods

### Plant Material

*Bergera koenigii* seeds were isolated from fresh fruit harvested from five trees. The seeds were peeled and placed under running tap water for 30 min, then shaken in 70% ethanol for 2 min. Alcohol-treated seeds were rinsed once with sterile distilled water, followed by immersion and shaking in 1.65% sodium hypochlorite supplemented with 1–2 drops of Tween-20 for 17 min. Finally, the seeds were rinsed with sterile distilled water three times and planted in tubes with solid MS medium (Murashige and Skoog, 1962) with 30 g l^–l^ of sucrose and 8 g l^–l^ agar. Surface-sterilized seeds were maintained in the dark for 5 weeks. After this period, germinated seedlings were exposed to constant white light at a fluence rate of 75 μmol m^−2^ s^−1^ for 3 weeks.

### Shoot Regeneration, Rooting, and Micrografting

Prior to transformation experiments, the optimal conditions for shoot regeneration and rooting were defined. Stems of *B. koenigii* seedlings were cut into 10–15 mm long explants and placed on solid MS medium supplemented with different formulations of plant growth regulators. Four replicate plates were set up per medium formulation treatment, with 12 explants per replicate. Subculture was performed at 2-week intervals. Shoots that sprouted from explants were counted after 4 weeks of cultivation. The following formulations of single growth regulators were used: 0.0, 1.0, 1.5, 2.0, 2.5, and 3.0 mg l^–l^ of benzyl-6-aminopurine (BAP); 0.0, 1.0, 2.0, and 3.0 mg l^–l^ for Kinetin (Kin); and 15.0, 25.0, and 35.0 mg l^–l^ for adenine sulfate (ADS). Four different combinations of BAP and naphthalene-acetic acid (NAA) were also tested (0 + 0 mg l^–l^; 1.5 + 0.5 mg l^–l^; 2.5 + 0.1 mg l^–l^; and 2.5 + 0.5 mg l^–l^ respectively). As browning and necrosis of stem explants occurred in all plates due to production of phenolic compounds in all cultures, the antioxidant polyvinylpyrrolidone (PVP) was added to the media at a concentration of 1 mg l^–l^ to reduce oxidation. The number of shoots produced per explant was determined after 4 weeks of culture.

The shoots produced were transferred to MS medium supplemented with different concentrations of auxins for *in vitro* root induction. The concentrations of added growth regulators were: NAA (0, 0.1, 0.5, and 1.0 mg l^–l^), indole-3-butyric acid (IBA), and indole-3-acetic acid (IAA; 0, 0.5, 1.0, and 2.0 mg l^–l^). The number of shoots producing roots was assessed after an additional 4 weeks of culture. As rooting was achieved at low levels for transgenic shoots, they were also micro-grafted into Carrizo citrus rootstock [*Citrus sinensis* (L.) Osbeck × *Poncirus trifoliata* (L.) Raf.] as described previously for transgenic citrus shoots (Orbović and Grosser, 2006). Rooted shoots were removed from Petri dishes and rinsed with distilled water to remove adhering agar. The plantlets comprised of both rooted and grafted shoots were transferred to pots covered with inverted magenta boxes to maintain high humidity and left on a light bench with constant white light (fluence rate of 50 μmol m^−2^ s^–l^). After 2 months in the laboratory, *B. koenigii* plantlets were transferred to the greenhouse, where they were acclimated to growth in larger pots.

### Vector Construct and Plant Transformation

The cDNA encoding Cry1Ba1 was cloned into a pCAMBIA2301 binary vector (NCBI accession AF234316) along with an N-terminal, 23 amino acid secretion signal derived from the snowdrop lectin, *Galanthis nivalis* agglutinin (GNA; Fouquaert et al., 2007), which promotes movement into the sieve element of the phloem, and the GFP coding sequence. First, the region of pCAMBIA2301 from the kanamycin resistance gene to *gus* (8,915–2,054 bp) was removed and replaced with the 35S promoter sequence, using primers P1-f and P1-r (Supplementary Table S1). The P1-f primer included *XhoI*, *EcoRI*, and *PstI* restriction sites with three nucleotides between each site for restriction digestion. These three restriction sites were added upstream of the 35S promotor sequence. The P1-r primer included *HindIII* and *SalI* restriction sites with three nucleotides in between, and these two enzyme restriction sites were added downstream of the 35S promoter sequence. These two primers also included 25 nt of sequence identical to sequence of the vector (2,055–8,914 bp) that was used for in-fusion cloning (Gibson Assembly, NEB, Ipswich, MA) to fuse the modified fragment with the vector backbone.

**Figure 1:**
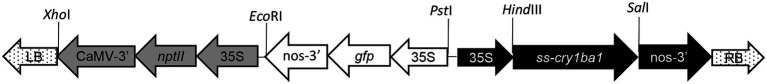
Schematic of T-DNA from the pCAMBIA2301-based binary vector pBa1 used for transformation of *B. koenigii* stem explants. Transcription of the CryBa1 coding sequence preceded by the GNA secretory signal (ss) was driven by the constitutive CaMV 35S promoter for expression in all plant tissues. Restriction sites used in construction of pBa1 are indicated.

The pCAMBIA2301 backbone (2,055–8,914 bp) was amplified using CloneAmp HiFi PCR premix (Fisher Scientific, Waltham, MA), with Backbone-f and Backbone-r primers (Supplementary Table S1). The purified vector backbone and insertion fragment described above were fused by using the In-Fusion HD Cloning Kit (Fisher Scientific, Waltham, MA). The recombinant vector called pPreBa1 was used for insertion of the following fragments: kanamycin resistance gene (neomycin phosphotransferase II; *nptII*) inserted into the *XhoI* and *EcoRI* sites; 35S-GFP-nos cassette from pLMNC95 (Mankin and Thompson, 2001) inserted into the *EcoRI* and *PstI* sites; and 69 nt GNA secretion signal (Fouquaert et al., 2007) and *cry1ba1* codon optimized for citrus—*Citrus x paradisi* (Nakamura et al., 2000; Shen et al., 2020) inserted into the *HindIII* and *SalI* sites to create the vector pBa1 (Figure 1). The *cry1ba1* sequence was comprised of the active, core sequence rather than the protoxin sequence such that proteolytic activation in the gut of the target insect was not required (Jurat-Fuentes and Crickmore, 2017).

To transform *B. koenigii,* we used a protocol routinely used for citrus transformation with slight modifications (Orbović and Grosser, 2015). Prior to the transformation experiment, an *Agrobacterium tumefaciens* culture harboring the pBa1 plasmid [EHA105 + pBa1] was prepared by growing a single colony in 50 ml YEP *Agrobacterium* cultivation medium containing 70 mg l^−1^ kanamycin and 50 mg l^−1^ rifampicin overnight at 28°C and 200 rpm, as described previously (Orbović and Grosser, 2015). A volume of 35 ml of the final culture was centrifuged at 3,000 x g for 10 min, and the pelleted bacteria was resuspended in 35 ml of co-cultivation medium, CCM (Orbović and Grosser, 2015). The OD600 was adjusted to 0.7 by addition of CCM medium.

*Bergera koenigii* cut stem explants were placed into liquid co-cultivation medium, CCM, for 2–3 h prior to co-cultivation with *Agrobacterium*. Explants were soaked in freshly prepared *Agrobacterium* suspension for 3 min, then incubated on plates with solid CCM medium for 2 days. As CCM has no antibiotics, *Agrobacterium* had 48 h to transform cells on the cut surfaces of explants. Explants were then transferred to regeneration medium [RM (Orbović and Grosser, 2015)] with the antibiotic cefotaxime (330 mg l^–l^) to eliminate *Agrobacterium* and kanamycin (70 mg l^–l^) to suppress the growth of non-transformed shoots. BAP (2.5 mg l^–l^) and NAA (0.5 mg l^–l^) were added to RM to promote shoot morphogenesis. Control shoots were regenerated from non-inoculated explants on RM medium without antibiotics. Shoots that sprouted from explants inoculated with *Agrobacterium* were inspected for GFP fluorescence under a fluorescence stereomicroscope Leica MZ16 FA (Leica, Bannockburn, IL) with a 480 nm excitation blue light.

### Confirmation of T-DNA Insertion and Transcription by Quantitative Real-Time PCR Analysis

To confirm integration of the T-DNA into the genome of *B. koenigii* plants with GFP fluorescence, DNA (~30 mg) was isolated from single leaves using the cetyltrimethylammonium bromide (CTAB) extraction procedure (Doyle and Doyle, 1987) with minor modifications. Briefly, tissue was pulverized in liquid nitrogen and transferred to a 2 ml tube containing 400 μl of 2xCTAB buffer (100 mM Tris-HCl pH 8.0, 1.4 M NaCl, 20 mM EDTA, 2% CTAB, 2% PVP, and 0.2% β-mercaptoethanol) and incubated at 60°C in a water bath for 30 min. After chloroform extraction, the aqueous phase containing the DNA was transferred to a new tube and subjected to RNAse A treatment at 37°C for 30 min. A second chloroform extraction was then conducted followed by phenol: chloroform extraction and isopropanol precipitation at −80°C for 30 min. The DNA pellet was washed with 75% ethanol and resuspended in 150 μl of H_2_O.

To confirm integration of *cry1ba* into the *B. koenigii* genome, the IDT PrimerQuest® tool (Owczarzy et al., 2008) was used to design three sets of primers at different positions in the Cry1Ba1 open reading frame (Supplementary Figure S1). The sequence of the three primers sets and internal control primer set of actin-2 (Harper et al., 2014) are provided in Supplementary Table S1. qPCR for the detection of *cry1Ba1* was conducted in 10 μl reactions in a 7,500 Thermal Cycler (Applied Biosystems, Waltham, MA, United States) using the Power SYBR™ Green RNA-to-C_T_1 step kit (Applied Biosystems Catalog # 4391178) without the RT-step. The 10 μl reaction consisted of 5 μl of 2XSYBR mix, 0.5 μl of 10 μm forward primer, 0.5 μl of 10 μm reverse primer, 0.1 μl SYBR enzyme, and 1 μl of template DNA diluted 1 to 50 to negate the effect of PCR inhibitors present in the resuspended DNA. The program was 95°C for 10 min to activate the polymerase followed by 40 cycles consisting of 95°C for 15 s and 60°C for 1 min. A melting curve was run at the end of qPCR program.

The transcription levels of *cry1ba1* in transgenic plants were estimated by RT-qPCR. Total RNA was extracted from *B. koenigii* leaves with the RNeasy Plant Mini Kit (Qiagen, Germantown, MD) and subjected to RT-qPCR analysis according to the manufacturer’s instructions. cDNA was synthesized from 1 μg of RNA using the iScript cDNA Synthesis kit (Bio-Rad Labs, Hercules, CA). ACT primers were used to amplify a fragment of the *actin* housekeeping gene as a reference for normalization of transcripts and the c*ry1ba1* target site was amplified using the primers, Ba1f and Ba1r (Supplementary Table S1). The qPCR validations were carried out using the iTaq Universal SYBR Green mix (Bio-Rad Labs, Hercules, CA) under the following conditions: 95°C for 2 min denaturation, 42 cycles at 95°C for 5 s, 57°C for 10 s, and 72°C for 15 s. Amplification specificity was verified by melt curve analysis from 55°C to 95°C. *cry1ba1* transcript levels in transgenic plants relative to WT were calculated using the 2^-ΔΔCt^ method (Livak and Schmittgen, 2001).

### Wilting Phenotype and Stomata Counts

Four non-transgenic (WT) and eight transgenic plants for which *cry1ba1* transcription was confirmed were used to examine the time to wilting. Two to three detached leaves were placed into Petri dishes (35 mm; Fisher brand, Thermo Fisher Scientific, Waltham, MA) containing a 1.5% agar bed. The agar in the dish maintained moisture (and leaf turgor) for approximately 2 weeks. The phenotype was recorded on day 3, day 7, and every 7 days thereafter and scored as 1-green, succulent; 2-green, dry; or 3-brown, wilted, dead, or dying in appearance. In addition, pinnate compound leaves from each WT and transgenic plant were detached and the stem inserted into a 1.5% agar solution placed in a 15 ml tube (Thermo Fisher Scientific, Waltham, MA). Due to the destructive nature of this approach, a single replicate was conducted for observation of the wilting phenotype.

To evaluate the numbers of stomata, leaves were removed from transgenic and WT *B. koenigii* (3 to 4 leaves per plant) and nail polish applied to the abaxial side. A 1 h later, tape was placed over the dried nail polish and slowly removed from the leaf surface. The stomatal impressions on the tape were observed under a microscope and the number of stomata determined per unit of surface area (0.042 mm^2^) for three different areas on each leaf, with 28 to 39 fields of vision in total per plant.

### Gas Exchange

The net gas exchange parameters, assimilation rate (of CO_2_), leaf transpiration rate, and stomatal conductance were determined with a LI-COR portable photosynthesis system (LI-6800; LI-COR Inc., Lincoln, NE). All measurements were taken in the morning from 10:00 to 11:30 am in the greenhouse. Photosynthetically active radiation (PAR) was set to 1,200 μmol m^−2^ s^–l^, leaf temperature was 32 ± 2°C, and vapor pressure deficit of 2.5 ± 0.4 kPa within the cuvette. Fully expanded leaves from three transgenic plants were used for these measurements.

### Phytohormone Analyses

The levels of different phytohormones in transgenic and control *B. koenigii* leaves were determined using gas chromatography–mass spectrometry (GC-MS). Multiple *B. koenigii* leaves were collected from each plant (wild type and transgenic plants 2, 6, and 16a), chopped, and combined for each treatment. Phytohormones were extracted as described by Nehela et al. (2016). Briefly, 0.1 g leaf tissue was ground into a fine powder using liquid nitrogen then mixed with 750 μl of extraction solvent (methanol: water: HCl; 80: 19.9: 0.1; v/v/v). The extraction procedure was repeated three times, and the supernatant concentrated to 50 μl under a gentle stream of nitrogen and stored at −80°C until use. Two derivatization procedures were employed as described previously (Nehela et al., 2016, 2018). Methyl chloroformate (MCF) was used to derivatize acidic phytohormones including three auxins [indole-3-acetic acid (IAA), IBA, and indole-3-propionic acid (IPA)], three salicylates [salicylic acid (SA), benzoic acid (BA), and *trans*-cinnamic acid (*t*CA)], *trans*-jasmonic acid [*t*JA], and abscisic acid [ABA]. *N*-Methyl-*N*-(trimethylsilyl) trifluoroacetamide (MSTFA) was used to derivatize the cytokinins [*trans*-zeatin (*t*Z) and *trans*-zeatin-riboside (*t*ZR)] and gibberellins [gibberellin A_3_ (GA_3_; gibberellic acid), gibberellin A_4_ (GA_4_), and gibberellin A_7_ (GA_7_)].

After derivatization, 1 μl of derivatized samples was injected into a Clarus 680 GC–MS (Perkin Elmer, Waltham, MA, United States) running in selected ion monitoring (SIM) mode using the thermo program and chromatographic conditions described previously (Nehela et al., 2016, 2018). Peaks were first identified by comparing mass spectra with library entries in the NIST Mass Spectral Library (National Institute of Standards and Technology, Gaithersburg, MA, United States) and Wiley Registry of Mass Spectral Data (9th edition; John Wiley and Sons, Inc., Hoboken, NJ, United States). Identification of all phytohormones was further confirmed by comparing retention time, linear retention indices (LRIs), and mass spectra with those of authentic standards. Typically, in the SIM-mode, three to five ions were monitored per compound and the abundance of those ions was comparable to those of the authentic standards (Nehela et al., 2016). Five technical replicates were performed for each transgenic and WT plant.

### Transmission Electron Microscopy of Asian Citrus Psyllids Fed on Transgenic and Control Plants

As western blot analysis for direct confirmation of Cry1Ba1 protein expression was confounded by the presence of high levels of phenolics in *B. koenigii*, an indirect approach to confirm pesticidal protein expression was adopted. Transmission electron microscopy was used to examine the guts of Asian citrus psyllids (ACP), *Diaphorina citri,* fed on transgenic *B. koenigii*, or WT plants, to look for gut damage associated with exposure to Cry1Ba1. A total of 20 adult ACP per plant were maintained on the plants for 8 days. A total of ≥40 live ACP adults were then collected and pooled per treatment for TEM analysis. The psyllids were dissected to remove the head and the tip of the abdomen, and then fixed for 16 h at 4°C in 1X PBS buffer containing 3% (v/v) glutaraldehyde, postfixed for 4 h at room temperature in 2% (v/v) osmium tetroxide, dehydrated in acetone, and embedded in Spurr’s Resin (Spurr, 1969). Light microscopy slides were prepared for orientation purposes to locate the midgut in cross section starting from the anterior or posterior ends of 8–10 psyllids from the WT and from each transformed plant. For TEM, 90 nm ultrathin sections were mounted on copper grids, stained with 2% (w/v) aqueous uranyl acetate and lead citrate (Reynolds, 1963), and observed using an FEI Morgagni 268 Transmission Electron Microscope (FEI Company, Hillsboro, Oregon, United States).

### Statistical Analysis

Shoot regeneration, rooting data, photosynthetic parameters, and phytohormone levels were analyzed for statistical significance using ANOVA. The Duncan’s multiple range test (DMRT) at *α* = 0.05 was used for comparisons between treatment means. For phytohormone analyses, *post-hoc* pairwise comparisons between the four test plants were performed with the Tukey-Kramer honestly significant difference test (Tukey HSD). Statistical significance was established at *p* < 0.05.

## Results

### Optimal Hormone Formulations for Shoot Regeneration and Rooting

The best individual plant growth regulator treatment for inducing shoot morphogenesis from stem explants was the 2.5 mg l^–l^ BAP treatment (Table 1). At that concentration of BAP, the mean number of shoots sprouting per explant was 2.15 ± 0.23 (SE). Both lower and higher concentrations of BAP induced fewer shoots. Explants cultivated on the medium supplemented with Kin in the range 1–3 mg l^–l^ exhibited shoot morphogenesis similar to that of explants cultivated on plates without Kin (Table 1). The addition of ADS resulted in increased shoot morphogenesis at 25.0 mg l^–l^, while ADS concentrations of 15 mg l^–l^ and 35 mg l^–l^ had no statistically significant effect (Table 1). NAA had no effect on shoot morphogenesis when added alone (data not shown). Based on our experience with *Murraya paniculata* (Shen et al., 2013), we also tested the effect of combinations of BAP and NAA on shoot morphogenesis and found the best response, 2.31 ± 0.35 shoots per explant, on medium supplemented with 2.5 mg l^–l^ of BAP and 0.5 mg l^–l^ of NAA (Table 1). This formulation was used for all subsequent experiments. Explants co-incubated with *Agrobacterium* showed reduced shoot morphogenesis on 2.5 mg l^–l^ BAP + 0.5 mg l^–l^ NAA at 0.89 ± 0.04 shoots per explant for material collected in the early part of the season (August–January). Seedlings germinated from seeds extracted from fruit harvested late in the season (February–March) had an even lower shoot regeneration rate at 0.59 ± 0.02 shoots per explant.

**Table 1:** Optimization of plant growth regulator content for induction of shoot morphogenesis from *Bergera koenigii* stem explants.

BAP	Mean ± SE	Kin	Mean ± SE	ADS	Mean ± SE	Medium formulation		Mean ± SE
						BAP	NAA	
0.0	0.22 ± 0.06 E	0.0	0.09 ± 0.03 A	0.0	0.06 ± 0.02 B	0.0	0.0	0.13 ± 0.08 B
0.5	1.02 ± 0.13 D	1.0	0.22 ± 0.06 A	15.0	0.13 ± 0.04 B	1.5	0.5	0.65 ± 0.14 B
1.0	1.33 ± 0.17 CD	2.0	0.21 ± 0.06 A	25.0	0.34 ± 0.08 A	2.5	0.1	1.52 ± 0.30 A
1.5	1.65 ± 0.21 BC	3.0	0.08 ± 0.04 A	35.0	0.09 ± 0.04 B	2.5	0.5	2.31 ± 0.35 A
2.0	2.00 ± 0.19 AB							
2.5	2.15 ± 0.23 A							
3.0	0.93 ± 0.15 D							

*Numbers of shoots per explant are shown. All growth regulator concentrations presented as mg l^–l^. Values accompanied by the same letters are not significantly different (DMRT). BAP, benzyl-6-aminopurine; Kin, Kinetin; ADS, adenine sulfate; and NAA, naphthalene-acetic acid*.

The percentage of shoots that grew roots after cultivation on different rooting media was low at up to 0.94% (Table 2). Improved rooting was only recorded on medium supplemented with NAA (Table 2). All shoots used in this series of experiments were harvested from explants co-incubated with *Agrobacterium*.

**Table 2:** Impact of type and concentration of auxins on rooting of shoots produced from *B. koenigii* stem explants.

NAA	Mean ± SE	IBA	Mean ± SE	IAA	Mean ± SE
0.0	0.06 ± 0.04 B	0.0	0.25 ± 0.09 A	0.0	0.06 ± 0.04 A
0.1	0.86 ± 0.17 A	0.5	0.17 ± 0.06 A	0.5	0.00 ± 0.00 B
0.5	0.94 ± 0.18 A	1.0	0.33 ± 0.08 A	1.0	0.11 ± 0.07 AB
1.0	0.64 ± 0.15 A	2.0	0.25 ± 0.08 A	2.0	0.19 ± 0.10 AB

*Percentage of shoots that grew roots are shown. All auxin concentrations presented as mg l^–l^. Values accompanied by the same letters are not significantly different (DMRT). NAA, naphthalene-acetic acid; IBA, indole-3-butyric acid; and IAA: indole-3-acetic acid*.

### Genetic Transformation and Confirmation of Cry1Ba1 Gene Insertion and Transcription

**Figure 2:**
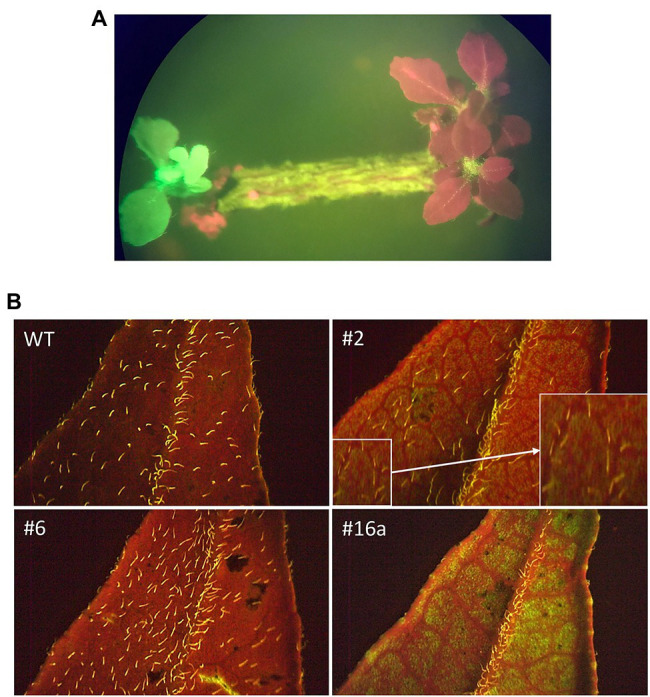
GFP fluorescence in transgenic *B. koenigii*. GFP fluorescence was used as a marker for identification of transgenic plants. **(A)** Explant with transgenic shoot with GFP fluorescence. The red shoots are non-transgenic. Transgenic shoots were selected on the basis of green fluorescence. **(B)** Sections of leaves from WT and transgenic plants 2, 6, and 16a. The fluorescence of trichomes on the plant surface can be seen in the WT image. GFP detection in leaf sections became more variable as the plants matured: Green fluorescence is evident for plant 16a, to a lesser degree for plant 2 (amplified image shown in inset), but not for plant 6. All images taken under blue light.

Transgenic shoots, identified on the basis of GFP fluorescence, were transferred to fresh media plates while still on the explant for the first 4–6 weeks. After this period, putatively transgenic shoots were cut from the explants and placed directly on medium where they grew at a very slow rate. Transformation rate was low at 0.89% (51/5705 shoots). This number includes 13 chimeric and 38 fully transformed shoots. Only fully transformed shoots were rooted or grafted. As the shoots grew into well-developed plants, the intensity of GFP fluorescence was reduced and varied between plants (Figure 2).

The integration of the *cry1ba1* construct into the *B. koenigii* genome was confirmed by use of qPCR with three sets of primers for transgenic plants 2, 6, and 16a but not control plants (Table 3). As the Ct values for the three distinct *cry1ba1* primer sets were 1 to 2 cycles below that of the actin-2 gene, multiple insertions are likely present in the *B. koenigii* genome. Relative transcription levels of *cry1ba1* in transgenic plants ranged from 35 (plant 23) to 538 (plant 16a; Table 4). Plants 2, 6, and 16a were selected for further analysis based on these relative transcription results.

**Table 3:** Confirmation of *cry1ba1* integration in transgenic plants.

	Primer set-1		Primer set-2		Primer set-3		ACT	
Plant	Ct	Tm	Ct	Tm	Ct	Tm	Ct	Tm
H_2_O	U	NA	U	NA	U	NA	U	N/A
WT1	U	NA	U	NA	U	NA	27.00 ± 0.38	76.06 ± 0.11
WT2	U	NA	U	NA	U	NA	26.48 ± 0.23	75.81 ± 0.19
2	24.76 ± 0.09	73.61 ± 0.11	24.54 ± 0.06	74.31 ± 0.19	24.41 ± 0.05	74.49 ± 0.19	26.14 ± 0.14	75.43 ± 0.19
6	24.31 ± 0.01	74.12 ± 0.19	24.28 ± 0.07	75.06 ± 0.00	24.59 ± 0.25	74.99 ± 0.11	26.70 ± 0.44	75.68 ± 0.11
16A	25.38 ± 0.13	74.31 ± 0.00	24.93 ± 0.02	75.24 ± 0.00	25.90 ± 0.04	75.06 ± 0.00	26.66 ± 0.07	75.81 ± 0.00

**qPCR analysis with three sets of primers (*Supplementary Table S1; Supplementary Figure S1*) confirming integration of cry1ba1 in transgenic B. koenigii lines 2, 6, and 16a. Data for two control plants (WT) and the qPCR negative control (H_2_O) are also shown. U, undetermined; NA, Not applicable**.

**Table 4:** Relative Cry1Ba1 transcription levels.

Plant	Relative transcript level
WT	0.0
2	461.0
6	158.0
16a	537.9
20	43.9
23	34.6
35	282.7

*Relative transcript levels of cryba1 in transgenic B. koenigii plants calculated with reference to actin using the 2^–ΔΔCt^ method*.

### Delayed Wilting Phenotype of Cry1Ba1 Transgenic Plants

**Figure 3:**
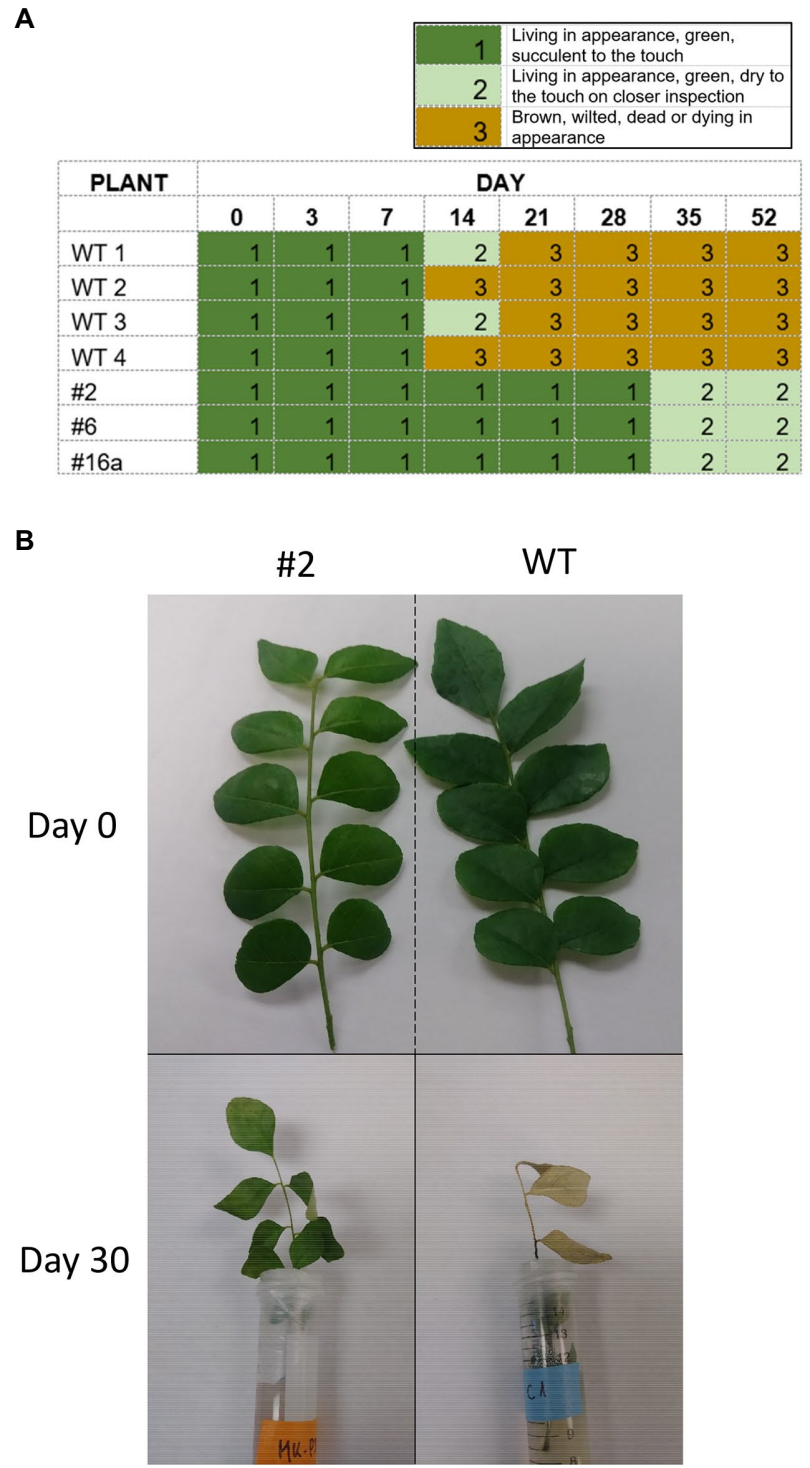
Delayed wilting phenotype of Cry1Ba1 transgenic plants. **(A)** Graphic showing the delayed drying of transgenic leaves maintained individually on agar in Petri dish assays, relative to WT leaves that had all dried and turned brown by day 21. **(B)** Representative images of transgenic Cry1Ba (plant 2) and WT *B. koenigii* leaflets taken at detachment (day 0) and after 30 days standing in vials with agar (day 30).

While handling leaves for experimentation, it was noted that detached leaves from wild-type plants wilted more rapidly than those from transgenic plants. In contrast to leaves from WT plants, which started to brown by day 14, leaves from the transgenic plants 2, 6, and 16a did not turn brown either when individual leaves were maintained in Petri dishes for 52 days or when leaflets were maintained in vials (Figure 3). The transgenic leaves eventually lost moisture and were dry to the touch (by day 35 in the Petri dish assay) but did not appear to have senesced in the traditional sense and did not exhibit browning and curling of the leaves.

### Alteration of Photosynthesis Parameters in Some Transgenic Plants

Measurements of photosynthesis parameters showed that transgenic plant 2 had stomatal conductance, CO_2_ assimilation rate, and evapotranspiration rate similar to those of WT plants (Table 5). Only transgenic plant 16a exhibited significantly higher CO_2_ assimilation rates than plant 2 and the WT plant, with plant 6 showing intermediate rates (Table 5). Plant 6 had a reduced stomatal density relative to WT and transgenic plants 2 and 16a (Table 5). As the number of stomata did not vary much between the transgenic plants (other than plant 6), a higher stomatal conductance was expected for the leaves that exhibited a high photosynthetic rate. That expectation was confirmed; plant 16a had significantly higher stomatal conductance than the WT and transgenic plant 2, while plant 6 exhibited an intermediate rate. These stomatal conductance rates were within the range previously recorded for citrus plants (Orbović et al., 2001). Similarly, the evapotranspiration rates from leaves of plants 16a and 6 were high and intermediate, respectively, relative to those of plant 2 and the WT plant (Table 5).

**Table 5:** Photosynthetic parameters in transgenic and wild-type *B. koenigii* leaves.

Plant	Assimilation rate (μmol m^−2^ s^−1^)	Number of stomata	Stomatal conductance (mmol m^−2^ s^−1^)	Transpiration rate (mmol m^−2^ s^−1^)
WT	1.66 ± 0.16 B	32.8 ± 1.0 A	7.67 ± 0.84 B	0.18 ± 0.01 B
2	1.23 ± 0.32 B	30.4 ± 0.9 A	6.38 ± 2.02 B	0.13 ± 0.04 B
6	2.65 ± 0.58 AB	26.6 ± 1.0 B	15.05 ± 2.92 AB	0.32 ± 0.04 AB
16a	4.09 ± 0.71 A	30.6 ± 1.1 A	29.92 ± 9.80 A	0.57 ± 0.21 A

*Data are presented as means ± SE; *n* = 6 except for stomata counts where *n* = 28–39. Values accompanied by the same letters are not significantly different (DMRT)*.

The results from the analyses of phytohormones extracted from leaves of WT and transgenic plants are presented in the Table 6. Phytohormone levels were quantified per unit of fresh weight. The following molecules were detected in leaves of all tested plants in similar quantities: *trans*-cinnamic acid (tCA), *trans*-jasmonic acid (tJA), indole-3-acetic acid (IAA), indole-3-propionic acid (IPA), IBA, gibberellic acid (GA3), and gibberellin A_7_ (GA7). The level of benzoic acid (BA) was 2759.1 ± 699.9 ng g^–l^ (mean ± SE) in the WT leaves, but much lower in transgenic plants ranging from 1093.7 ± 138.1 ng g^–l^ to 1662.7 ± 433.5 ng g^–l^. The same trend was recorded for salicylic acid (SA), which was detected at a high level in WT leaves 19523.0 ± 971.0 ng g^–l^ but ranged from 17434.7 ± 1106.0 ng g^–l^ to 17972.0 ± 499.1 ng g^–l^ in the leaves of transgenic plants. Significantly lower levels of abscisic acid (ABA) were detected in transgenic leaves (ranging from 260.0 ± 23.6 ng g^–l^ to 271.7 ± 20.8 ng g^–l^) in comparison with WT leaves (342.0 ± 11.8 ng g^–l^; DMRT). Finally, gibberellin A4 (GA4) was also detected in higher amounts in WT plants (32.7 ± 3.2 ng g^–l^) than in transgenic plants (from 26.0 ± 3.0 ng g^–l^ to 26.3 ± 2.5 ng g^–l^). In contrast, trans-zeatin-riboside (tZR) and especially trans-zeatin (tZ) were detected at higher levels in transgenic plants than in WT plants. Levels of tZ ranged from 21.2 ± 2.0 ng g^–l^ to 27.4 ± 2.5 ng g^–l^ in transgenic plants and 15.8 ± 1.5 ng g^–l^ in WT. The tZR was detected at 25.3 ± 8.5 ng g^–l^ in transgenic plant 2 in comparison with 14.4 ± 2.5 ng g^–l^ in the WT plant. Although the levels of tZR were also higher in transgenic plants 6 and 16a than in the WT, those differences were not statistically significant (Tukey HSD).

**Table 6:** Plant hormone levels in transgenic and wild-type *B. koenigii*.

Hormone	Hormone levels (mean ± SE) ng g^−1^			
*Plant*	WT	2	6	16a
tCA	12622.3 ± 1313.4 A	11322.0 ± 1103.6 A	11801.3 ± 1340.0 A	10796.7 ± 1585.9 A
**BA**	2759.1 ± 699.9 A	**1093.7 ± 138.1 B**	**1241.3 ± 200.9 B**	**1662.7 ± 433.5 B**
**SA**	19523.0 ± 971.0 A	**17434.7 ± 1106.0 B**	17972.0 ± 499.1 AB	**17625.7 ± 95.5 B**
tJA	546.7 ± 101.9 A	506.0 ± 56.3 A	397.3 ± 47.9 A	460.0 ± 103.0 A
IAA	183.7 ± 59.8 A	150.0 ± 44.7 A	148.3 ± 20.3 A	158.3 ± 11.8 A
IPA	370.3 ± 26.7 A	334.3 ± 43.8 A	300.0 ± 20.1 A	291.0 ± 84.1 A
IBA	306.3 ± 35.9 A	244.7 ± 56.9 A	242.7 ± 22.6 A	298.0 ± 26.6 A
**ABA**	342.0 ± 11.8 A	**268.3 ± 47.2 B**	**260.0 ± 23.6 B**	**271.7 ± 20.8 B**
**tZ**	15.8 ± 1.5\u00B0C	**27.4 ± 2.5 A**	**21.2 ± 2.0 B**	**24.0 ± 2.1 AB**
**tZR**	14.3 ± 2.5 B	**25.3 ± 8.5 A**	23.6 ± 3.6 AB	23.6 ± 2.1 AB
GA3	39.7 ± 4.0 A	40.3 ± 5.0 A	37.7 ± 3.1 A	39.7 ± 2.1 A
**GA4**	32.7 ± 3.2 A	**26.3 ± 2.5 B**	**26.3 ± 1.5 B**	**26.0 ± 3.0 B**
GA7	64.0 ± 4.6 A	63.0 ± 4.6 A	60.0 ± 9.8 A	58.3 ± 1.2 A

*Hormones concentrations detected in B. koenigii leaves using GC-MS-SIM are shown. Values significantly different from WT are indicated in bold (Tukey HSD). tCA, trans-Cinnamic acid; BA, Benzoic acid; SA, Salicylic acid; tJA, Jasmonic acid; IAA, Indole-3-acetic acid; IPA, Indole-3-propionic acid; IBA, Indole-3-butyric acid; ABA, Abscisic acid; tZ, trans-Zeatin; tZR, trans-Zeatin riboside; GA3, Gibberellic Acid; GA4, Gibberellin A_4_; and GA7, Gibberellin A_7_*.

### Cry1Ba1-Mediated Damage to ACP Gut Tissue

**Figure 4:**
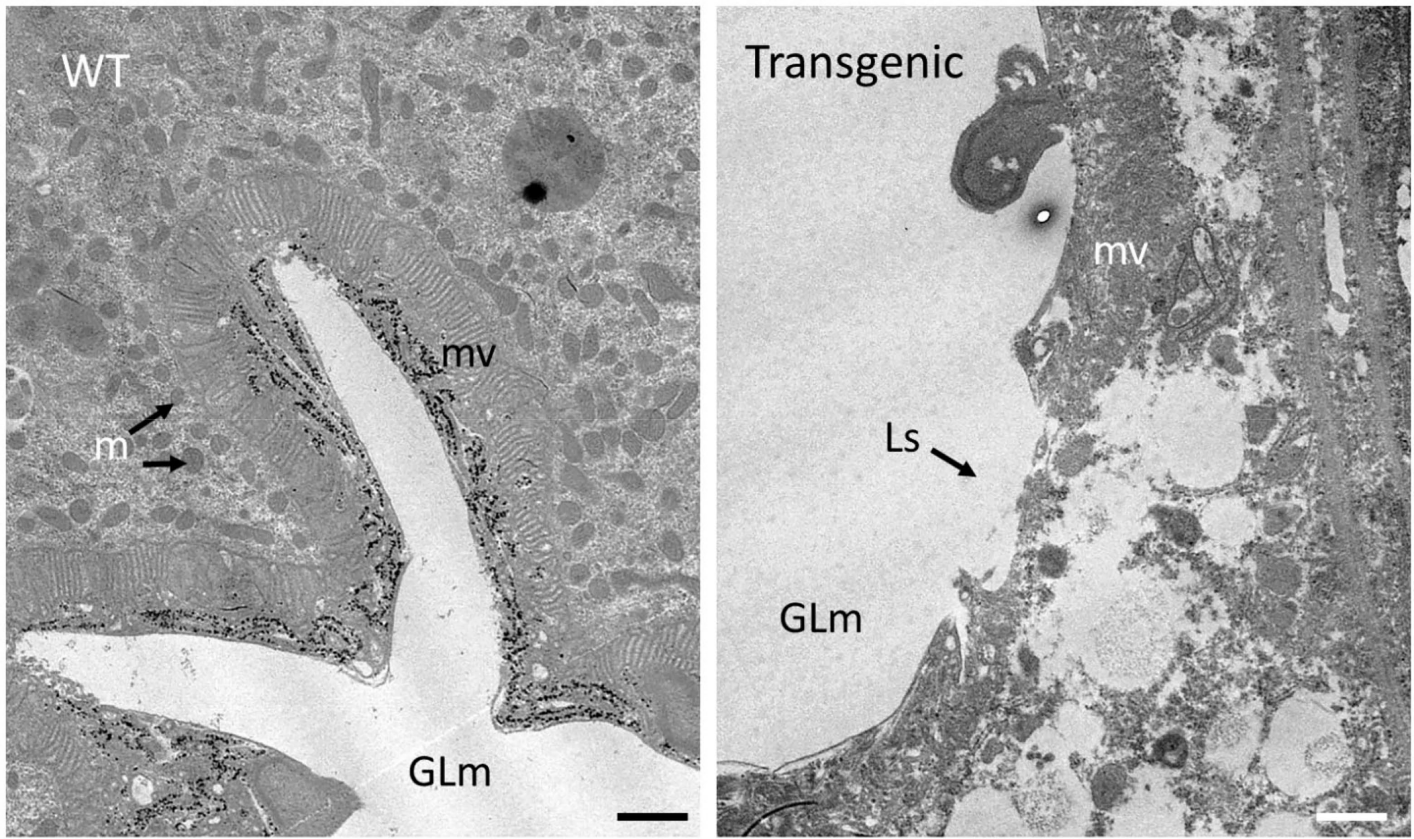
Cry1Ba1-mediated damage to the gut epithelial tissues of Asian citrus psyllid. Transmission electron micrographs showing representative images of the gut epithelium of ACP fed on a wild type (WT) or transgenic plant. The intact microvillar lining of the gut epithelium is evident in ACP fed on WT plants. In contrast, the microvilli of insects fed on Cry1Ba1-expressing *B. koenigii* (Transgenic) were sparse and disrupted with multiple lesions apparent. These micrographs confirm transgenic plant expression of Cry1Ba1. GLm, gut lumen; mv, microvilli; m, mitochondria; and Ls, lesion in microvilli. Bar, 1 μm.

Guts dissected from ACP fed on either wild type or Cry1Ba1 transgenic plants were examined by transmission electron microscopy. While uniform microvilli were consistently observed lining the gut epithelial cells of ACP fed on WT plants, extensive lesions were observed in the microvilli lining the gut of ACP fed on the Cry1Ba1 plants (Figure 4). Such lesions and disruption of the insect gut epithelium are typical of damage caused by three-domain Cry pesticidal proteins such as Cry1Ba1. This damage to the ACP gut epithelium confirms expression of the active core of Cry1Ba1 from the transgenic plants.

## Discussion

Here, we report genetic transformation of *B. koenigii* for the first time and demonstrate expression of both GFP and the bacteria-derived pesticidal protein, Cry1Ba1. This result paves the way for production of transgenic *B. koenigii* for use as trap plants for suppression of ACP populations within an integrated pest management framework.

### Optimal Formulation for Shoot Regeneration Defined

Plants of this species can be propagated by seeds and vegetative cuttings, although this method requires use of high concentration of IBA to induce rooting (Kathik and Joshi, 2014a). Previous work to optimize *in vitro* micropropagation of *B. koenigii* concluded that the presence of the cytokinin BAP was crucial for successful shoot regeneration and IBA was crucial for rooting of shoots (Rani et al., 2010, 2012; Kathik and Joshi, 2014a,b). Similar conclusions were drawn for conditions defined for *in vitro* propagation of the close relative of *B. koenigii*, the orange jasmine plant, *Murraya paniculata* (Shen et al., 2013). In the present study with *B. koenigii*, while BAP significantly promoted shoot morphogenesis at 2.5 mg l^–l^, no impact was seen upon addition of IBA to the rooting medium. Differences between *B. koenigii* used in the respective studies in India and in the United States could contribute to the different outcomes for IBA. Shoots that sprouted from explants used in our transformation experiments grew very slowly and responded poorly to auxins added to the medium to induce rooting.

### Challenges Posed by High Phenolic Levels

Seedling explants used for our transformation experiments produced high levels of phenolic compounds that needed to be controlled by the addition of PVP. Indeed, high levels of polyphenolics posed technical challenges for DNA and protein extraction from plant material, and for their transfer to membranes for blotting. Despite success with citrus varieties, western blots and Southern blots with *B. koenigii* material were hampered, and alternative approaches were used to confirm T-DNA integration into transgenic plants (PCR) and Cry1Ba1 production by transgenic plants (TEM of psyllids to look for Cry1Ba1-mediated damage to the gut epithelium).

### Cry1Ba1 Impacts Plant Physiology

The transformation rate for *B. koenigii* was low at 0.89%. The transgenic plants produced had a lower growth rate than the WT plants (data not shown), but other morphological traits were similar to WT plants. When considering photosynthesis measurements, transgenic plants 6 and 16 were expected to have droopy or wrinkled leaves due to their higher evapotranspiration rates and higher stomatal conductance (Table 5). In contrast, detached leaves from all transgenic plants wilted significantly more slowly than those from WT plants. Leaf senescence is controlled by various environmental and internal signals that are modulated by the levels of plant hormones (Woo et al., 2019). We therefore addressed whether plant hormone levels in *B. koenigii* transgenic plants differed from those in WT plants.

Hormonal analyses of samples from transgenic and WT *B. koenigii* plants showed that cytokinins tZ and tZR were present at higher levels in leaves of transgenic plants, whereas BA, SA, ABA, and GA4 were present at lower levels (Table 6). With respect to their involvement in leaf senescence, ABA (Sah et al., 2016), SA (Rivas-San Vicente and Plasencia, 2011), and jasmonic acid (JA; Zhu et al., 2015) are major promoters of senescence. It is widely accepted that cytokinins are involved in maintenance of photosynthetic activity and the delay of senescence (Wingler et al., 1998). All promoters of senescence except JA were present in leaves of transgenic *B. koenigii* plants at lower levels and inhibitors at higher levels. These results suggest that transgenic plants are experiencing endogenously induced stress related to the production of Cry1Ba1 and respond by activating processes that can decelerate senescence and/or cell death. This response in our transgenic plants is the result of the interaction of gene networks regulated mostly by the cytokinins ABA and SA, but not JA.

The levels of GA_4_ (one of four naturally active gibberellins) were also lower in transgenic plants. The role of gibberellins in leaf senescence is not clearly defined (Woo et al., 2019), but they are known to regulate stem elongation (Achard and Genschik, 2009). Reduced GA_4_ in transgenic plants may account for their slower growth rate (data not shown). Lower levels of BA in leaves of transgenic plants could be tied to the role of SA in the process of senescence because BA is a direct precursor molecule in the synthesis of SA. Other phytohormones that were quantified in leaves were present in similar levels in all plants (Table 6). The levels of ABA were equally depressed in all transgenic plants (Table 6); this was not reflected in the regulation of stomatal aperture (Table 5). The higher stomatal conductance and evapotranspiration recorded in transgenic plants 6 and 16 were not supported by lower levels of ABA in plants 6 and 16 alone, which points to a role for other processes affecting the cell/leaf physiology of the transgenic plants. Future transcriptomic analysis of leaf tissue from transgenic plants would provide greater insight into the effect of Cry1Ba1 on plant cells. Notably, *in planta* expression of a number of bacteria-derived pesticidal proteins has proven similarly challenging, and the underlying impacts on plant physiology are not yet defined. Challenges associated with *in planta* expression have limited the commercial utility of some bacterial pesticidal proteins in some crop plants.

### Use of Trap Plants for ACP Management

Additional tools beyond insecticidal sprays are needed for management of ACP in citrus groves. The use of transgenic plants expressing Bt-derived pesticidal proteins would reduce insecticide inputs and align well with practices used by citrus growers in integrated pest management programs. For the delivery of Bt pesticidal proteins, it would be beneficial to use a plant other than citrus that is attractive to ACP but not a host for CLas, such as *B. koenigii* (Beloti et al., 2017, 2018). Transgenic *B. koenigii* that produces Bt pesticidal proteins could be planted around the perimeter of citrus groves to attract ACP, which would ingest the pesticidal protein and die before reaching the citrus trees. A similar strategy employed in citrus orchards in Brazil met with some success, where non-transformed *M. paniculata* was planted around citrus trees and sprayed with insecticides. The number of ACP that flew into the orchards through the gaps between sprayed *M. paniculata* trees was significantly lower relative to orchards where trap trees were not planted (Tomaseto et al., 2019). Most recently, it was reported that *B. koenigii* is also the preferred host for *Triozae erytreae* Del Guercio, the insect vector for *Candidatus* Liberibacter africanus, that results in HLB in Africa (Aidoo et al., 2019). The use of transgenic plants expressing pesticidal proteins such as that described here could provide an additional preventative tool for HLB management in citrus on a global scale.

## Conclusion

Transgenic curry leaf trees, *Bergera koenigii*, producing the bacteria-derived pesticidal protein Cry1Ba1 were generated. Integration of the Cry1Ba1 coding sequence into the *B. koenigii* genome and transcription from the inserted cassette were confirmed. In contrast to Asian citrus psyllids fed on wild-type plants, those fed on transgenic plants suffered damage to the gut epithelium consistent with the toxic action of Cry1Ba1. This damage provides indirect evidence for *in planta* expression of Cry1Ba1. As curry leaf trees are highly attractive to ACP, transgenic *B. koenigii* resistant to ACP has potential for use as trap plants around citrus groves as an additional tool for ACP management. Demonstration of the production of transgenic *B. koenigii* is the first step toward this goal.

## Data Availability Statement

The original contributions presented in the study are included in the article/Supplementary Material. Further inquiries can be directed to the corresponding author.

## Author Contributions

BB, CE-M, LS, NK, SR, and VO designed the experiments. DA, LS, NK, SR, TS, VO, and YC performed the experiments and analyzed the data. VO and BB wrote the manuscript. All authors contributed to the article and approved the submitted version.

## Funding

This project was supported by the Citrus Diseases Research and Extension grants program, Award number 2017-70016-26755 and by the Emergency Citrus Diseases Research and Extension grants program, Award number 2020-70029-33177 from the USDA National Institute of Food and Agriculture.

## Conflict of Interest

The authors declare that the research was conducted in the absence of any commercial or financial relationships that could be construed as a potential conflict of interest.

## Publisher’s Note

All claims expressed in this article are solely those of the authors and do not necessarily represent those of their affiliated organizations, or those of the publisher, the editors and the reviewers. Any product that may be evaluated in this article, or claim that may be made by its manufacturer, is not guaranteed or endorsed by the publisher.
