# Supplementary material for: Genetic Modification of Bergera koenigii for Expression of the Bacterial Pesticidal Protein Cry1Ba1
Source: Front Plant Sci. 2022 May 24;13:899624. doi: 10.3389/fpls.2022.899624 (PMC9171844; doi:10.3389/fpls.2022.899624)
Supplement: Supplementary file 1 [file Data_Sheet_1.pdf]

Supplementary Material

TABLE S1. Primers used for PCR in this study. Restriction sites are underlined.

**Name**	**Sequence**	
Vector Construction		
P1 (f)	5'-CTACTCACACATTATTATGGAGAAACTCGAGaaaGAATTCaaaCTGCAGGGCACTGGCCGTCGTTTTACAACGTCGTGACTG-3'	
P1 (r)	5'-GGAAATTCGAGCTGGTCACCTGTAATGTCGACTATAAGCTTAGTCCCCCGTGTTCTCTCCAAATGAAATGA-3'	
Backbone (f)	5'-GAGTTTCTCCATAATAATGTGTGAGTAG-3'	
Backbone (r)	5'-ATTACAGGTGACCAGCTCGAA-3'	
Transgenic plant insert analysis		***Position in cry1ba1***
PS-1F	TGGTTGCTTTGTTTCCTTCTTAC	764-786
PS-1R	CCAGTAGCTCCAATAGCATCAG	863-842
PS-2F	CTACAGAGAGGCCTAACTACGA	1334-1355
PS-2R	GCTGATCTGTGTGTCCAAGAA	1442-1422
PS-3F	CTCAGTACATTGCTCTTGAATTGG	431-454
PS-3R	AGATTAGCAGCCTGAGCATAAA	536-551
ACT-F	GTTGCCATTGGTTGGTATTTGATAC	N/A
ACT-R	CGTCGACTGCCATTCCAGAT	N/A
Transcript abundance analysis		
ACT (f)	5'-TCACAGCACTTGCTCCAAGCAG-3'	
ACT(r)	5'-TGCTGGAAGGTGCTGAGGGA-3'	
Ba1(f)	5'-GCCTATTCACGGAGTTCCAA-3'	
Ba1(r)	5'-CAACTGAAGACCTGGTGATTC-3'	

FIGURE S1. Codon-optimized Cry1Ba1 coding sequence. The annealing sites for the three sets of primers used for insert confirmation (Table S1) are indicated: Primer set 1: bold, underlined; Primer set 2: white text, gray shaded; Primer set 3: bold, italics. The 69 nt sequence encoding the GNA secretory signal are indicated in red.

ATGGCTAAGGCTTCTCTTTTGATTTTGGCTGCTATTTTTCTTGGAGTTATTACTCCTTCATGTTTGTCTGAAGAT
TCACTTTGTATTGCTGAGGGTAATAACATTGATCCATTTGTTTCAGCTTCTACTGTTCAAACAGGAATTAATATT
GCTGGTAGAATTCTTGGAGTTTTGGGTGTTCCTTTTGCTGGACAGCTTGCTTCTTTTTACTCATTTTTGGTTGGA
GAACTTTGGCCAAGGGGTAGGGATCAATGGGAGATTTTTCTTGAACATGTTGAGCAGCTTATTAATCAACAGATT
ACAGAAAACGCTAGGAATACTGCTCTTGCTAGATTGCAAGGACTTGGAGATTCTTTTAGGGCTTATCAACAGTCA
TTGGAAGATTGGCTTGAGAATAGAGATGATGCTAGAACAAGATCAGTTTTGTACA***CTCAGTACATTGCTCTTGAA***
TTGGATTTTCTTAACGCTATGCCTCTTTTTGCTATTAGGAATCAAGAGGTTCCACTTTTGATGG***TTTATGCTCAG***
GCTGCTAATCTTCACCTTTTGCTTTTGAGAGATGCTTCTTTGTTTGGATCAGAATTTGGTCTTACATCTCAAGAG
ATTCAGAGATATTATGAAAGACAAGTTGAGAGAACTAGGGATTACTCAGATTACTGTGTTGAATGGTACAACACA
GGATTGAACTCTCTTAGGGGTACTAATGCTGCTTCATGGGTTAGATACAACCAGTTTAGAAGAGATCTTACATTG
GGTGTTCTTGATT**TGGTTGCTTTGTTTCCTTCTTAC**GATACTAGGACATATCCAATTAATACTTCAGCTCAGCTT
ACAAGAGAGGTTTATA**CTGATGCTATTGGAGCTACTGG**TGTTAACATGGCTTCTATGAATTGGTACAATAACAAT
GCTCCTTCATTTTCTGCTATTGAAGCTGCTGCTATTAGATCACCACATCTTTTGGATTTTCTTGAGCAACTTACA
ATTTTCTCAGCTTCATCTAGGTGGTCAAATACTAGACATATGACATATTGGAGGGGACACACTATTCAGTCTAGA
CCTATTGGTGGAGGTTTGAATACTTCTACACATGGTGCTACTAACACATCAATTAATCCAGTTACACTTAGATTT
GCTTCTAGGGATGTTTATAGAACTGAATCATATGCTGGAGTTCTTTTGTGGGGTATCTATCTTGAGCCTATTCAC
GGAGTTCCAACAGTTAGGTTTAATTTCACTAACCCTCAAAACATTTCTGATAGAGGAACAGCTAACTATTCTCAA
CCTTATGAATCACCAGGTCTTCAGTTGAAGGATTCAGAAACTGAGCTTCCTCCAGAAACTACAGAGAGGCCTAAC
TACGAGTCATACTCTCATAGATTGTCTCACATTGGTATTATTCTTCAGTCAAGAGTTAACGTTCCAGTTTATTCT
TGGACACACAGATCAGCTGATAGAACTAATACAATTGGACCTAACAGGATTACTCAAATTCCAATGGTTAAGGCT
TCAGAATTGCCTCAGGGTACTACAGTTGTTAGAGGACCAGGTTTTACTGGTGGAGATATTCTTAGAAGGACTAAT
ACTGGTGGTTTTGGACCTATTAGAGTTACAGTTAATGGTCCATTGACTCAAAGATACAGGATTGGTTTTAGATAC
GCTTCTACAGTTGATTTCGATTTCTTTGTTTCAAGAGGTGGTACTACAGTTAATAACTTCAGATTTTTGAGGACT
ATGAATTCAGGAGATGAGCTTAAGTACGGTAACTTCGTTAGAAGGGCTTTTACTACACCTTTTACTTTTACACAA
ATTCAGGATATTATTAGAACTTCTATTCAGGGTCTTTCAGGAAATGGTGAAGTTTATATTGATAAAATTGAGATT
ATTCCATAA
